# Polygenic Scores in the Direct-to-Consumer Setting: Challenges and Opportunities for a New Era in Consumer Genetic Testing

**DOI:** 10.3390/jpm13040573

**Published:** 2023-03-23

**Authors:** Jin K. Park, Christine Y. Lu

**Affiliations:** 1 Harvard Medical School, Boston, MA 02115, USA; 2 Department of Population Medicine, Harvard Medical School and Harvard Pilgrim Health Care Institute, Boston, MA 02115, USA; 3 Kolling Institute, Faculty of Medicine and Health, The University of Sydney and the Northern Sydney Local Health District, Sydney, NSW 2077, Australia; 4 School of Pharmacy, Faculty of Medicine and Health, The University of Sydney, Sydney, NSW 2006, Australia

**Keywords:** polygenic risk scores, direct-to-consumer genetic tests

## Abstract

Direct-to-consumer (DTC) genetic tests have generated considerable scholarly attention and public intrigue. Although the current consumer genetic testing regime relies on the reporting of individual variants of interest to consumers, there has recently been interest in the possibility of integrating polygenic scores (PGS), which aggregate genetic liability for disease across the entire genome. While PGS have thus far been extensively explored as clinical and public health tools, the use of PGS in consumer genetic testing has not yet received systematic attention, even though they are already in use for some consumer genetic tests. In this narrative review, we highlight the ethical, legal, and social implications of the use of PGS in DTC genetic tests and synthesize existing solutions to these concerns. We organize these concerns into three domains: (1) industry variation; (2) privacy and commercialization; and (3) patient safety and risk. While previously expressed concerns in these domains will remain relevant, the emergence of PGS-based DTC genetic tests raises challenges that will require novel approaches.

## 1. Introduction

Direct-to-consumer genetic tests allow consumers to access genetic testing without the direct involvement of healthcare providers [1,2]. By 2025, the global DTC genetic testing market is projected to be worth upwards of 2.5 billion dollars [3]. One of the most important technological innovations generating widespread interest from academics, clinicians, and consumers alike is the incorporation of polygenic scores (PGS) for consumer genetic testing. While many initially expressed skepticism that PGS could ever be ready for widespread use, many commentators have also argued that it was only a matter of time before the use of PGS in the DTC setting became widespread [2]. Indeed, PGS-based DTC genetic tests are now here [4]. Although significant academic efforts are underway to understand the utility of PGS as clinical or public health tools, the use of PGS in consumer genetic testing has not received the same attention. 

In this narrative review, we highlight the ethical, legal, and social implications of the use of PGS in consumer genetic testing and review various suggestions as to how these concerns may be addressed. We focus primarily on the implications of the use of PGS in common diseases of adult-onset (i.e., type 2 diabetes, cardiovascular disease, Alzheimer’s, breast cancer, and others), though the use of PGS-based genetic testing for embryo selection and prenatal testing poses important challenges of their own, some of which may overlap with those discussed here. We organize these concerns into three domains: (1) industry variation; (2) privacy and commercialization; and (3) patient safety and risk. Instead of taking a definitive stance on the immense complexities discussed below—particularly given the ongoing academic and industry efforts being brought to bear on improving the efficacy of polygenic risk scoring—this review seeks to synthesize existing scholarship and encourage scholarly and public engagement. 

## 2. Polygenic Scores: An Overview

Many previous models of DTC genetic tests were largely confined to monogenic testing or non-health-related ancestry evaluations. In contrast, the polygenic score is the latest iteration of a series of advances in genomics that has the potential to expand prevailing understandings of the scope and role of DTC genetic testing. Although some of the literature uses “polygenic risk score” and “polygenic score” interchangeably, we follow conventions that reserve the latter term to extend to a broader array of measures that may include rare as well as common variants [5], and that can be applied in traits where “risk” may not be applicable [6]. In many contexts, however, both terms are often used to refer to the score calculated by taking a weighted sum of the effect sizes of disease-associated Single Nucleotide Polymorphisms (SNPs) from Genome-Wide Association Studies (GWAS). There are, of course, methods to explain the genetic basis of phenotypic variation without exclusively relying on SNPs—the most common source of human genetic variation. Although the PGS creates a statistic to estimate an individual’s susceptibility for diseases based on variants across the genome, there are still limitations to their clinical and individual efficacy [7]. 

Regardless, the use of PGS in DTC genetic tests is already here. In 2019, 23andMe^®^ unveiled a PGS-based consumer report for type 2 diabetes. Myriad Genetics and Genomics PLC have incorporated PGS into their clinical risk models [8,9]. PGS-based tests are also available for more exploratory uses; for example, PGS-based tests are also being offered during pre-implantation genetic testing of embryos for certain common diseases such as diabetes and cancer [10]. As the applicability of PGS expands, a deeper question at the heart of modern consumer genomics that has increasingly come into focus is this: what is consumer genomics for? Broader scholarly engagement on this critical question will be important as efforts are underway to determine professional, regulatory, and ethical standards for the use of PGSs in the DTC setting.

Published literature on PGS and related issues has understandably been dominated by the use of PGS in clinical settings. Although some of the implications of PGS-based consumer genetic tests will overlap with previous concerns that were raised in the advent of consumer genetic testing, the entry of polygenic scoring creates unique challenges that warrant further research and broader public and scholarly engagement (Table 1). 

## 3. Industry Variation: Two Steps Forward, One Step Back for Consumer Genetic Testing?

One of the most pressing issues for the widespread use of PGS in DTC settings is industry variation. There are at least three dimensions of industry variation that are relevant: (1) variation in the calculation of PGS; (2) variation in the ability of companies to improve and update PGS models with additional mechanistic and functional information; and (3) variation in the generation of consumer risk reports. We will consider each in turn.

### 3.1. Industry Variation in PGS Modeling 

Firstly, company-specific data analysis strategies drive the calculation of PGS. At a general level, virtually all models for the calculation of PGS using GWAS data involve at least two steps: (1) a selection of the SNPs that contribute to the model and (2) a selection of the weights that apply to each SNP [11]. While there are now many open-source algorithms for the calculation of PGS, there is still significant room for variability in choosing optimally performing PGS given specified parameters [7,9]. As reviewed by Wang et al., investigators are also developing novel approaches to improve PGS accuracy by aggregating GWAS summary statistics across different traits and genetic ancestry groups [9,12].

The differences underlying various strategies for PGS generation have important consequences. A recent study that compared recently published PGS for three common diseases using the UK Biobank discovered that only 80% of the SNPs in one PGS were included in the other, leading to diverging risk stratification between the two models [13]. Furthermore, although the discovery samples used to obtain SNPs and their effect sizes may be available from publicly accessible GWAS data, companies may perform their own GWAS to calculate PGS for their own target population, and validate it with publicly accessible GWAS data when available [14]. This is, for instance, how 23andMe^®^ has explained their strategy for calculating PGS for type 2 diabetes [15]. In addition, as reviewed by Witte et al., the contribution of individual variants to the underlying genetic basis of various diseases can differ based not only on the underlying genetic architecture of a disease, but also the scaling used to assess heritability [16]. 

Secondly, industry leaders, due to their market share, possess greater amounts of data to improve the accuracy of proprietary models for various target populations. The development of methods and data collection efforts to improve the predictive ability of PGS across populations will continue, requiring vigilant efforts to close the gap in predictive power of PGS across consumers of diverse genetic ancestries [17,18,19]. 

Lastly, there is the additional source of industry variation that comes from the assessment and reporting of genetic disease risk from PGS. Companies may choose to report either absolute risk (i.e., the probability that an individual will develop a given disease in their lifetime) [20] or relative risk (which some have argued is misleading for explaining the benefits of PGS in some contexts) [21]. In either scenario, proprietary methods may play a significant role in disease reporting. This challenge hearkens back to the difficulties surrounding significant variation in risk estimation for the same disease based on first-generation consumer genetic testing years ago; to assess industry-wide consistency in the past, entities such as the Government Accountability Office could send identical saliva samples to DTC companies and assess the comparative validity of risk estimates for monogenic disorders [22]. If PGS are widely utilized in DTC genetic testing, however, it is unclear whether regulators or the general public can easily ascertain what proportion of the inconsistency in risk estimation can be attributable to algorithmic differences in the platform (Table 1). 

### 3.2. Industry Standards for PGS-Based DTC Genetic Tests 

Adopting certain standards for the reporting of PGS could partly allay these concerns. Scholars have recently sought to articulate reporting standards for PGS as research tools, including standards that may be important first steps for clinical and analytical validity as clinical tests compliant with the Clinical Laboratory Improvement Amendments [23,24]. Indeed, Wand et al. discuss considerations such as risk model predictive ability, positive/negative predictive value, and risk model interpretation among others as important for PGS reporting going forward, especially as efforts are made to translate them to the clinic [23]. PGS standardization efforts will surely be critical for assessing industry variation in the DTC genetic testing marketplace, where standardized reporting conventions for heritability, model prediction, and risk attribution may help make more direct comparisons between PGS models. Although academic efforts are underway to create universal reporting standards for the development, performance, and applicability of PGS—with these efforts driven in part by the need for such standards for PGS to pass regulatory muster—it is unclear whether these reporting standards will be adopted by industry partners without external regulation or consumer demand. As scholars have argued, regulatory agencies such as the United States Food and Drug Administration (FDA) may be uniquely positioned to promote data sharing in this area, as well as transparency and industry standardization [25,26]. 

Others may argue instead for industry self-regulation. Experts have noted the ongoing trend of consumer genetic testing companies pursuing mergers and partnerships with hospital organizations to move away from a DTC model to a “hybrid model” [27,28]. If trends continue, one may wonder whether the alarm over PGS in DTC settings may be obviated in the long term. However, others have argued that industry self-regulation has thus far not shown itself to be sufficient for the proper governance of PGS in DTC settings, seen most prominently in the case of PGS being offered in embryo-selection and for pre-implantation genetic testing for certain common diseases such as diabetes and cancer [29]. Although the use of PGS in pre-implantation genetic testing has been banned in countries such as the United Kingdom, in the United States, it has largely gone unregulated [10]. Professional bodies such as the American College of Medical Genetics have recommended against offering PGS-based prenatal testing in the DTC setting given that “genetic studies on complex traits and disease constitute an inexact science and do not identify affected individuals as does testing for monogenic disorders” [30]. In addition to the complexity of traits, there are additional ethical concerns for the use of PGS in prenatal testing that must be considered [31]. It is unclear whether industry leaders will adopt the recommendations of professional bodies regarding PGS-based consumer genetic testing. 

At the same time, the DTC genetic testing industry has developed rapidly in the past decade. During the expansion of mass consumer genetic testing, there was widespread concern that the lack of industry standards may require robust regulatory intervention [32]. Today, however, the presence of well-established industry leaders such as 23andMe^®^, Ancestry, and others may allow for more effective adoption of scientific reporting standards for PGS if these standards are developed. 

## 4. Privacy and Commercialization in the Context of Widespread PGS-Based Consumer Genetic Testing

Genomic privacy has emerged as a key area of public and scholarly concern in the literature. The increasing rate of consumer participation in DTC genetic testing has created large databases storing consumer genetic information, raising new ethical, legal, and social questions regarding privacy, data security, and commercial genetics. Because more extensive treatments of privacy in the genomic era can be found elsewhere [33,34], here we discuss three issues raised in the literature regarding PGS and SNP profiles: (1) the use of SNP profiles in contexts uncoupled to its original purpose; (2) third-party genetic interpretation services; and (3) the scope of commercial data use. 

Although privacy has long been a concern for human genetic information [33], experts have recently highlighted new concerns related to the use of genetic information from newer genetic tests to serve other purposes. For instance, it has been suggested that the use of SNP profiles for a diverse set of law enforcement-related ends poses novel legal questions, especially in the context of increased genetic sequencing [33,34,35,36]. Accordingly, patients’ source of concern regarding the privacy of their genetic information is diverse, and more research is needed into sociodemographic differences in individuals’ motivation for seeking genetic testing, as well as their concerns around data sharing for other purposes [35] (pp. 12–15), especially given industry variation in privacy practices [28,33]. 

Relatedly, PGS-based DTC genetic tests also raise important questions about the structure of life insurance schemes. Because population-wide risk stratification is a routine practice of underwriting insurance policies tailored to segments of the population, some have speculated whether PGS-based genetic information may offer a natural extension for existing insurance schemes [37]. Furthermore, if PGS use becomes sufficiently widespread and accepted, it is unclear how insurance underwriters will treat high polygenic disease liability itself as a risk factor [38]. 

Data privacy is an especially important concern with PGS due to the rise in third-party interpretation services for genetic information. Though some consumers that choose to upload their genetic information to third-party interpretation services have been satisfied with the results, there are also important concerns regarding consistency and validity of results across interpretation services [28,39]. As mentioned in the previous section, industry variation in reporting of PGS and their associated risk of disease may be exacerbated given the increased use of third-party genetic interpretation services, many of which have thus far fallen outside of FDA oversight [28,40]. Because third-party interpretation services and DTC genetic testing firms are often not affiliated with healthcare institutions, they are typically not a “covered entity” regulated by the Health Insurance Portability and Accountability Act of 1996 [33,40], though they may be subject to more expansive state-level privacy regulations [27]. There have been efforts to understand consumers’ motivations for seeking third-party interpretation services, as well as to compare the existing landscape of third-party interpretations services [40,41,42]. For example, Nelson and Fullerton found that some third-party interpretation services that provide PGS based on consumers’ data conceive their tool as a “bridge” to the scientific literature without making clear medical or clinical recommendations [42]. 

Lastly, the emergence of PGS in DTC tests also raises important new questions for commercial responsibility regarding data use, such as the limits (if any) to the iterative use of patient SNP profiles to improve the accuracy of proprietary algorithms. Consumers have expressed worries about genetic testing companies selling their data to unauthorized third parties, a key concern that has emerged in parallel debates regarding data protection policies among social media companies [43]. Given that PGS are currently most relevant as research tools, even if consumers are not provided disease-specific PGS, there is a question of how patients’ data from research participation should be returned, as well as ensuring that the scope of data use is included during the provision of informed consent [38]. Consumer genetic testing companies such as 23andMe^®^ contract with pharmaceutical companies to create more effective drug development pipelines, even if there is no direct exchange of identifiable consumer data. When consumers agree to participate in large-scale genomics research in pooled databases, questions regarding the reach of these informed consent agreements may be important to consider, as well as other innovative proposals for informed consent such as a dynamic consent model (Table 2) [44,45]. 

Though there is extensive literature on the sharing of information discovered from DTC genetic tests to family members, it is far less clear how PGS-based DTCs will change dynamics around responsibility for sharing of genetic information to family members. Recent work has demonstrated that depending on specified thresholds, high polygenic scores for certain traits may be correlated across siblings [46]. Yet, because PGS aggregate the effect sizes of many common variants across the genome, depending on the genetic architecture of a condition, consumers without a family history of that condition may nonetheless receive a high PGS [47]. It is therefore unclear what principles should guide the communication of PGS-based genetic information to family members, and further research in this area is warranted. 

**Table 2:** Stakeholders and Potential Solutions for Key Issues in PGS-based Consumer Genetic Testing.

Concern	Potential Solutions	Key Stakeholders
Lack of portability of PGS for consumers of diverse ancestral backgrounds	Increase diversity of study populations and data collection efforts responsibly to improve effect size estimates and other metrics. Build co-constitutive partnerships with underserved communities historically neglected in scientific research [6,19,38,48].	Researchers, consumers, citizens
	Develop multi-ethnic PGS that are portable across different populations and ancestral backgrounds [9,12,17]	Researchers
	Improve public data sharing and transparency efforts to promote replicability of existing PGS while accelerating the development of reliable trans-ethnic PGS across diseases [14,23,38]	Researchers, regulators
Variation in firm-specific reporting of PGS and their associated risks (if any)	Adapted from Wand et al [23]: Researchers should closely adhere to various efforts to standardize the reporting of PGS such as the Polygenic Risk Score-Reporting Standards (PRS-RS) which include information regarding study population, methods used to develop and evaluate the risk model, limitations on generalizability, and data transparency	Researchers
	Conduct studies to compare the performance of various PGS for similar study populations and conditions [13,24]	Researchers
	Develop robust frameworks that can integrate PGS with other conventional risk factors (if relevant) that will allow PGS to be used to stratify patient cohorts in relation to overall risk [11,16,20,23]	Researchers, regulators, healthcare providers, industry partners
	Report industry variation to relevant regulators as well as firm-specific quality control mechanisms	Consumers, healthcare providers
	Develop ethical, professional, and scientific standards for reporting of genetic basis for wider range of non-disease-related traits apart from ancestry [27,28,30,38]	Researchers, healthcare providers
	Foster broader public understanding of genomic risk in a new era of PGS-based genetic tests	Consumers, researchers, healthcare providers, regulators, industry partners
Commercial data use and genetic privacy	Bolster existing regulatory regimes at federal, state, and local levels to inform consumers of the benefits and risks of sharing their samples for genetic testing [28,32,36]	Regulators, industry partners, researchers, consumers, healthcare providers
	Further research on the correlational structure of PGS for family members across traits and thresholds, adopt thresholds and standards for relevance of data to family members [29,38,46]	Researchers, healthcare providers, consumers
	Study existing data use agreements, promote greater transparency and individualized motivations for seeking testing, and consider novel approaches such as dynamic consent models for evolving research needs [33,35,44,45]	Consumers, regulators, industry partners, researchers
Widespread use of third-party interpretation services	Develop standards for use of PGS such as the PRS-RS (Wand et al.) [23] by bolstering consumer choice, through academic efforts to compare various third-party interpretation services, and industry self-governance [23,40,42]	Consumers, healthcare providers, researchers
	Further establish regulatory standards for third-party interpretation services that will promote consumer safety and personal health [28,39,40,41,42]	Regulators, consumers, researchers
Comprehension of genetic risk and genetic determinism	Perform further research on the ability of PGS to promote patient comprehension and health in the context of conventional risk factors and lifestyle, additional research to replicate recent findings of patient health literacy and health-promoting behavior using PGS-based tools across disease contexts [30,49,50,51,52,53,54]	Researchers, consumers, healthcare providers
	Study the ability of consumers to understand genetic determinism and polygenic risk for disease in the context of PGS-based genetic tests across traits and disease conditions for themselves and for their family [21,38,46,55]	Researchers
	Explore whether regulatory avenues that create rules of the road for firms that report PGS-based tests are warranted [26,28,32,56]	Regulators, consumers, researchers
Provider self-efficacy of sharing and discussing PGS-based results	Develop professional guidelines for the clinical utility of PGS-based genetic tests for specific subpopulations, as well as limitations for specific subgroups [23,30,38,57]	Healthcare providers, researchers
	Empower genetic counselors, physicians, and other healthcare professionals with new tools to ensure that they can incorporate PGS into practice [58]	Healthcare providers
Possibility of stigma for PGS-based genetic results	Build robust systems of social support for genetic counselors, health professionals, patients, and their families to ensure that stigma can be minimized and genetic information can be used responsibly	Healthcare providers, researchers
	Perform further research on the possibility of psychological distress for new technology and expand stakeholders on particularly troubling worries of stigma for mental health [59,60]	Researchers, consumers

This table lists important concerns and possible solutions expressed in the literature, along with relevant stakeholders.

## 5. Patient Safety and Risk: A Renewed Discussion of the Proper Scope of Regulation in 21st Century Consumer Genomics?

### 5.1. Safety and Risk with PGS-Based Genomic Tools 

Safety is a key concern for any medical intervention, and a significant literature has developed regarding the safety profile of DTC genetic tests, though more work is needed on whether findings on previous models of genetic testing apply to newer models that incorporate PGS. There is conflicting evidence regarding the health-promoting or health-detrimental impacts of monogenic DTC genetic tests. While some studies have shown that access to genetic health information can encourage patients to take greater control of their health, particularly in the context of cancer-disposing gene mutations, other studies have shown less conclusive results [61,62,63]. Particularly, some have demonstrated that a false negative result in some cases might create an unjustified reassurance of health, whereas a false positive result can lead to “overtreatment” [61,64,65]. For instance, although 23andMe^®^’s breast cancer reports explicitly state that the three BRCA1/2 pathogenic variants being tested do not include other BRCA1/2 pathogenic variants relevant for breast cancer risk [4], it is unclear whether patient comprehension of incomplete results in this setting will translate to PGS-based tests. 

Polygenic scores throw a wrench into an already complex debate regarding genetic risk comprehension. First, PGS generally explain only a fraction of the heritability of a complex condition. Accordingly, some experts deflate the utility of PGS on this basis, arguing that other conventional risk factors may be more actionable. However, this is likely disease-dependent. For example, as reviewed by Wray et al., studies have demonstrated that PGS for some conditions (e.g., coronary artery disease and breast cancer) may already be a powerful tool for risk assessment, and in many cases improved the utility of existing risk assessment tools based solely on conventional risk factors [47]. Furthermore, studies have shown that PGS can pick out individuals with a risk for common diseases that is equivalent to some monogenic mutations [66]. In addition, part of the so-called “missing heritability” of some complex disorders may be discovered as more individuals are genotyped, and as more rare variants are queried in the process [67]. Accumulating functional evidence regarding variant–disease relationships may also improve the clinical utility of PGS, as well as their accuracy in diverse genetic ancestries [68]. 

Crucially, many scholars have urged the lack of generalizability of PGS across ethnicities, given that many large-scale genomics studies have been performed in European populations [6,69]. There are efforts underway to address this on both ends—by diversifying data collection efforts in a responsible manner and also by developing newer algorithms to improve the portability of PGS across genetic ancestries [9,12,17,19,48]. Some companies such as 23andMe^®^ have stated that their PGS-based tools are already accurate for individuals of non-European genetic ancestries [2,70], attributing this to their unique database of research participants, which purportedly includes “the most Latino/Hispanic and African American research participants of any genetic database in the world” [70]. Other DTC firms reportedly tailor certain genetic tests to individuals of specific ancestries [38]. Notably, 23andMe^®^ currently provides information to consumers regarding groups for whom information about a given variant is most useful. For example, for the Late-onset Alzheimer’s Disease risk report based on the *APOE* gene, 23andMe^®^ states that risk estimates may not be equally accurate across all ethnic backgrounds [1].

It is unclear, however, how this kind of approach should be tailored for PGS, where a complex interplay of factors may drive differences in accuracy of PGS for various groups, including (most prominently) lack of representation in genomic databases, differing allele frequencies, and others. 

Secondly, PGS-based stratification of consumers’ disease risks may impact consumer behavior. Although the several studies performed thus far on the impact of communicating polygenic disease risk on patient behavior revealed that polygenic risk may help to motivate positive health-seeking behavior in the context of cardiovascular disease [49,50,51], there has been particular concern regarding the use of PGS for certain psychiatric conditions, where polygenic risk information could expose consumers to additional stigma [59,60], as well as concern regarding the importance of avoiding genetic determinism [55,71]. However, a recent systematic review to determine the behavioral impact of sharing PGS discovered that high PGS in the bipolar disorder and breast cancer settings were associated with reduced feelings of self-blame and guilt, though there was significant variability among the studies [52]. Furthermore, a discrete choice experiment by Venning et al. found that consumers ranked test accuracy, applicability across cancer types, and testing through a general practitioner (rather than online) as important factors in a PGS-based test [53]. A qualitative study of the perspectives of a diverse group of patients on PGS also discovered that the majority of study participants preferred in-person disclosure of PGS results by a physician [54]. Further research is needed to confirm these findings, and determine whether PGS-based genetic risk can help or hinder patients’ understanding of their disease and how PGS-based results affect consumer behavior. 

Thirdly, consumers’ and health providers’ understanding of PGS and PGS-related results may be varied. Scholars have documented that while both specialist and non-specialist healthcare providers are generally better equipped than consumers to interpret traditional DTC genetic tests, there is a lack of research regarding whether providers’ self-efficacy may be equivalent for PGS-based genetic tests, or whether they believe PGS-based tests are practically relevant [57,58,72,73]. 

### 5.2. Regulatory Considerations 

Regulatory solutions to these safety concerns are important to consider. Although several federal agencies in the United States have taken a role in regulating consumer genetic testing in the past, it is unclear how the regulatory landscape will change in the face of widespread PGS-based consumer genetic testing and third-party interpretation. The Federal Trade Commission (FTC), US Centers for Medicaid & Medicare Services (CMS), and FDA have all played a role in overseeing consumer genetic testing [28,33]. The FTC is charged with protecting consumers against deceptive practices, and has noted the unique privacy concerns of consumer genetic testing [28], though it has infrequently taken direct action against DTC genetic testing companies [33]. To ensure high-quality laboratory testing for clinical analytes, CMS regulates laboratory tests through the Clinical Laboratory Improvement Amendments. 

The FDA regulates some DTC genetic tests as in vitro devices, but their oversight of DTC genetic testing has been varied. The first DTC tests approved for marketing by the FDA were from 23andMe^®^ [1]. Industry leaders, scholars, and FDA have demonstrated interest in striking a balance between protecting patient and consumer safety and preserving consumers’ access to certain forms of genetic health information. The emergence of PGS creates challenges and opportunities to strike this balance. Although there are many complex regulatory questions prompted by the rise of PGS-based genetic testing, particularly salient in this context is according to publicly available company offering documents, their PGS-based tool is a “low-risk general wellness product” [28]. 

Consistent with this claim, 23andMe^®^ seems to be taking an approach not completely out of line with the general wellness claims for type 2 diabetes contemplated by the FDA. According to one source, 23andMe^®^ allows consumers to see, for an imaginary pool of 100 individuals with PGS-based profiles similar to theirs, how the addition of risk factors such as weight and frequency of fast food consumption changes the probability of developing diabetes [2,4]. Indeed, at the clinical level, although PGS are unlikely to be used as the sole risk factor to guide management and treatment decisions, many have advocated the use of PGS as one tool among others (including patient history and other clinical/conventional risk factors) for stratifying the prevention and management of complex conditions. For example, investigators have developed risk models that include PGS to stratify patients in a clinically meaningful manner across a variety of disease contexts [11,47,74]. Similarly, PGS in the DTC setting may also be integrated with other risk factors to help to stratify consumers based on a comprehensive model of risk within which PGS is but one component (along with other clinical and lifestyle factors). For instance, Muse et al. have developed a smartphone application to do exactly this, integrating PGS along with other lifestyle factors to provide patients with overall clinical risk [50].

In one possible scenario, the question of whether the PGS-based tests are general wellness products may depend on how these other risk categories are reported, as well as what “risk cohort” a given patient falls into based on their PGS [2]. In the midst of a growing DTC genetic market, some developers for third-party interpretation services have indicated the need for “rules of the road”—specifically, the lack of clear direction from regulatory bodies means that developers and industry leaders are often working in the midst of regulatory ambiguity and without clear guardrails [42]. 

In many ways, the ambiguity around the regulation of PGS-based DTC genetic tests was anticipated, including limitations of some existing frameworks for the regulation of DTC genetic testing as the line between monogenic and polygenic disease paradigms quickly became blurred [56]. Indeed, existing frameworks for regulation such as the five-step model developed by Wright et al. are helpful starting points for consensus-building, even if concepts such as “biomarker-disease association” and “scientific validity” may need to be re-worked given the considerations we have reviewed above [32].

Further scholarship and robust dialogue are needed regarding whether/how the FDA should have oversight over these tools, given that immediate regulation may not be the answer to every problem in this space [63]. In the United States, more extensive interagency coordination between the FDA, FTC, and CMS may be appropriate as PGS-based tests become more widespread and technical challenges necessitate a more nimble approach [28]. As past experience has shown, balancing the interests of protecting consumers who interface with genetic health information with the population- and individual-level benefits that may be available as a result of our expanding knowledge of genomics will be a constantly moving target [38]. 

Over the long-term, more expansive non-regulatory solutions should also be considered. In many ways, PGS hold immense potential as a powerful tool in the DTC setting because it is the bridge between personalized and population medicine. Common variants and their effect sizes are difficult to understand in isolation, and PGS can only sensibly be estimated by aggregating findings from population studies. If the goal of DTC testing is to empower patients and to “democratize” medicine, then it will be important to understand the role of genomic information for individuals on their own terms given their unique circumstances, values, and preferences. 

## 6. Conclusions

The use of PGS in DTC genetic testing presents novel challenges that warrant further study and public involvement. We have highlighted three domains—industry variation; privacy, and commercialization; patient safety and risk—in which PGS-based testing will pose significant challenges and deviate from consumers’ previous engagement with DTC tests. While we did not perform a systematic literature search, we have carefully reviewed the published literature related to PGS since the initial advent of DTC genetic testing in the last decade and synthesized them into these domains. Research on PGS in DTC settings is in its nascent stages, and further studies on these domains is warranted, with a specific focus on quantifiable metrics to assess effects on consumer health-related behaviors.

Although it will be important to establish robust guidelines for the use of PGS in consumer genetic testing, previous scholarship will still be useful in setting standards for PGS-based tests. Therefore, though regulators and researchers need to start off on the right foot, they need not start from scratch. At the same time, PGS-based consumer genetic testing will also stretch the use of genomics by the public beyond existing thresholds, necessitating robust cross-disciplinary efforts to ensure the safety and efficacy of these technologies. Novel participatory solutions to bolster public understanding of genomic technology should also be considered to empower consumers in efforts to democratize genomic technology safely and effectively. 

Some have argued that in the near future, as genomic testing gains currency as consumer products for a “price conscious, genetically literate, and busy world”, a significant portion of the public will have access to their whole genome sequences in a portable and mobile format [57]. Indeed, if consumers rank convenience highly and drive demand for DTC genetic testing to meet these expectations, it will be important for clear parameters to be established for PGS and other summary scores. Insofar as scientific and clinical actors aim to support demands for accessible consumer genetic testing within the guardrails of safety and efficacy, improved standards and regulation are needed to minimize adverse effects, many of which we have discussed in this paper. While industry variation, privacy, data protection, and safety concerns related to discrepancies in reporting and interpretation are clear challenges, there will surely be others (Table 2).

More broadly, if empowering the general public to have agency over their genetic information is a key value for all stakeholders, then promoting increased genetic literacy will be a key bulwark (along with appropriate regulatory and industry standards) for protecting the interests of consumers. Deliberative forums in which experts, industry leaders, and most importantly—citizens and consumers—are convened to debate and understand the purpose and function of PGS-based technologies may be critical to empower the general public to understand these tools going forward, and can help to ease the transition if they begin to be incorporated into clinical systems. For instance, deliberation in citizens’ assemblies have recently been proposed to help resolve governance questions for thorny ethical issues arising in the context of other emerging technologies such as germline genome editing [75]. Therefore, a crucial component of the next few years will be to involve a wide-ranging group of participants—citizens in particular—to ensure that the public can participate in a robust and transparent discussion about the purpose and responsible use of genomic technology.

## Figures and Tables

**Table 1:** Comparison Between Two Types of DTC-GTs.

Domain	Theme of Comparison	Individual Variant-Based DTC Testing	PGS-Based DTC Testing
Industry variation	Scope of genetic information	Narrow, centered on evidence-based variants with significant evidence	Significant variation may range from PGS as one component among others, or possibly a more expansive role for PGS and summary scores
	Variation based on genetic ancestry	Individual variants of special concern in particular populations often noted	Lower predictive power of PGS for some populations
	Reporting standards	Significant variation initially, standardization following GAO-led investigation and academic studies	Significant variation likely based on firm-specific algorithms, with unclear regulatory/market impetus
	Non-disease-related conditions	Some health-related genetic information provided (e.g., ancestry)	PGS-based genetic liability for non-disease-related traits feasible often with unclear evidence base
Privacy and commercialization	Privacy	Genetic discrimination, consent procedure, data security and protection	Similar worries, with additional concerns about consumer consent for iterative data use, investigative genetic technology using SNP profiles
	Third-party interpretation	Third-party interpretation discussed when FDA initially sent regulatory intent letters to US genetic testing firms, and some firms released raw data	Third-party interpretation likely widely offered and utilized
	Commercialization	General concerns around market share, consumer protection	Additional concerns about the use of consumer data for proprietary research and other purposes, and how they compete with privacy protection
	Sharing to family members	Significant concerns around informing family members, warnings generally issued to consumers	No guidance for relevance of PGS for family members, PGS for family members shown to be correlated in some scenarios
Safety and risk	Patient comprehension	Worries around genetic determinism and false reassurance of health	Similar worries, with the added complication of probabilistic inference
	Provider comprehension	Relative confidence in healthcare providers’ self-efficacy of interpreting DTC-based genetics with patients	Limited studies, further investigation warranted
	Stigma	Mixed evidence in the literature about stigma and psychological impact of genetic information disclosure	Expressed worries about stigmatizing effects of PGS-based information for psychiatric conditions
	Regulatory framework	Certain device-associated medical claims regulated by FDA with some FTC and state-level involvement	Limited studies, further investigation warranted into how existing regulatory regimes can be adapted

This table highlights important differences and similarities in two types of direct-to-consumer genetic testing that are discussed in this review, and is thus not meant to be exhaustive. GAO, Government Accountability Office; FDA, Food and Drug Administration; DTC, direct-to-consumer; FTC, Federal Trade Commission; SNP, Single Nucleotide Polymorphism.

## Data Availability

This study did not report any data.
